# Final pressure setting of programmable valve in ventriculo-atrial shunt for idiopathic normal pressure hydrocephalus

**DOI:** 10.1186/2045-8118-12-S1-O49

**Published:** 2015-09-18

**Authors:** Kiyoshi Takagi

**Affiliations:** 1 Chiba-Kashiwa Tanaka Hospital, Japan

## Introduction

A quick reference table for setting programmable pressure valves in patients with idiopathic normal pressure hydrocephalus (iNPH) has been proposed for ventriculo-peritoneal shunt (VP shunt) (Miyake et al. Neurol Med Chir (Tokyo) 48, 2008). Recommended pressure has strong correlation with body mass index (BMI). However, little is known about the ideal pressure setting for ventriculo-atrial shunt (VA shunt). The purpose of this paper is to show the final pressure setting in the iNPH patients with good outcome received VA shunts and to investigate the correlation between the final pressure setting and preoperative factors.

## Patients and Methods

Eighty-four iNPH patients with good outcome (improved modified Rankin Scale , improved mini-mental state examination over 3, cease of urinary incontinence, or reduced care-giver's burden without improvement of modified Rankin Scale) at one year after VA shunts using programmable valve with anti-siphon device were the candidates of this study. Correlations between final pressure setting and BMI, body length, body weight, and preoperative cerebrospinal fluid (CSF) pressure measured by lumbar tap were investigated. Data were shown in mean +/- SD and statistically analyzed by calculating Pearson product-moment correlation coefficients and the significant level was set at p less than 0.05.

## Results

Mean age was 77.6 +/- 6.1 years old (male : female = 48 : 36). Mean body length, body weight, and BMI were 157.2 +/- 8.8 cm, 54.1 +/- 10.8 kg, and 21.8 +/- 3.3 respectively. Preoperative CSF pressure was 118.9 +/- 34.2 mmH2O. The mean initial valve pressure setting was 126.3 +/- 17.6 mmH2O (median = 120 mmH2O) and the mean final pressure setting was 62.1 +/- 31.3 mmH2O (median = 55 mmH2O). There were no significant correlations between the final pressure setting and BL, BW, BMI, and CSF pressure. In 43 cases, final pressures were below 50 mmH2O including 23 cases with the lowest setting of 30 mmH2O.

## Discussion and conclusion

This study clearly demonstrated that there were no preoperative determinants for the ideal setting of valve pressure in VA shunt. It also demonstrated that the final setting was unexpectedly low and it suggests the necessity of lower setting valve.

